# Oxford Nanopore MinION Direct RNA-Seq for Systems Biology

**DOI:** 10.3390/biology10111131

**Published:** 2021-11-04

**Authors:** Mikhail A. Pyatnitskiy, Viktoriia A. Arzumanian, Sergey P. Radko, Konstantin G. Ptitsyn, Igor V. Vakhrushev, Ekaterina V. Poverennaya, Elena A. Ponomarenko

**Affiliations:** 1 Institute of Biomedical Chemistry, 119121 Moscow, Russia; arzumanian.victoria@gmail.com (V.A.A.); radko@ibmc.msk.ru (S.P.R.); konstantin157@yandex.ru (K.G.P.); vakhrunya@gmail.com (I.V.V.); k.poverennaya@gmail.com (E.V.P.); 2463731@gmail.com (E.A.P.); 2 Federal Research and Clinical Center of Physical-Chemical Medicine, 119435 Moscow, Russia

**Keywords:** transcriptomics, nanopore technology, RNA-seq, MinION, pathway activation, systems biology, HepG2

## Abstract

**Simple Summary:**

A new technology has been recently developed by Oxford Nanopore Technologies, enabling researchers to investigate the structure and relative abundance of specific molecules, ribonucleic acids. The ribonucleic acids carry information from the genes to proteins, which are responsible for virtually every process in the human organism, including disease progression and response to therapies. Special computational methods allow identification of various activated biological processes by analyzing the changes in concentrations of ribonucleic acids. This is of particular interest for precision medicine which aims at single-patient analysis. Here we evaluated whether ribonucleic acid abundances measured by new technology are suited for robust predictions of activated biological processes in single samples. We performed simulations varying the number of experimental replicates and analysed activated biological processes’ predictions using two algorithms. In brief, we found that at least two replicates are required to obtain reproducible results. We hope that our findings may be of interest to researchers planning their nanopore experiments and may stimulate further development of clinical applications of this technology.

**Abstract:**

Long-read direct RNA sequencing developed by Oxford Nanopore Technologies (ONT) is quickly gaining popularity for transcriptome studies, while fast turnaround time and low cost make it an attractive instrument for clinical applications. There is a growing interest to utilize transcriptome data to unravel activated biological processes responsible for disease progression and response to therapies. This trend is of particular interest for precision medicine which aims at single-patient analysis. Here we evaluated whether gene abundances measured by MinION direct RNA sequencing are suited to produce robust estimates of pathway activation for single sample scoring methods. We performed multiple RNA-seq analyses for a single sample that originated from the HepG2 cell line, namely five ONT replicates, and three replicates using Illumina NovaSeq. Two pathway scoring methods were employed—ssGSEA and singscore. We estimated the ONT performance in terms of detected protein-coding genes and average pairwise correlation between pathway activation scores using an exhaustive computational scheme for all combinations of replicates. In brief, we found that at least two ONT replicates are required to obtain reproducible pathway scores for both algorithms. We hope that our findings may be of interest to researchers planning their ONT direct RNA-seq experiments.

## 1. Introduction

Transcriptome analysis aims to provide information about the complete set of RNA transcripts in the body under certain conditions. This type of molecular profiling can be used to quantify gene expression and capture alternative splice variants. In contrast with the genome, which is mostly static, the transcriptome reflects the dynamic nature of complex regulatory interactions between genes and proteins.

While microarrays have been the most popular platform for gene expression profiling through the 2000s, the introduction of RNA-seq technology in 2008 offered several significant advantages, including higher dynamic range, low background signal, and ability to detect novel splice variants and mutations [1]. The short-read RNA-seq technology has become a “gold standard” for gene expression quantification providing an in-depth understanding of biological processes [2]. Furthermore, RNA-seq depletion protocols from Illumina were found to work well with degraded and low-quantity samples, which is a major advantage for potential clinical applications [3]. However, short reads are less suitable for sequencing long transcripts, thus increasing the likelihood of mapping errors for isoform identification [4].

A new era was opened in transcriptome analysis with the introduction of long-read or third-generation sequencing. While conventional short-read “RNA-seq” is rather inappropriately termed so since the RNA molecules are not directly sequenced [5], long-read sequencing deals with full-length native RNA molecules without the necessity to perform reverse transcription or amplification [6]. Direct RNA sequencing allows detection of nucleotide modifications and estimation of poly-A tails length [7,8]. In addition, it has significant advantages in identifying alternative splicing isoforms eliminating the need for read mapping or assembly. Spike-in experiments have shown that direct RNA sequencing can accurately quantify gene and isoform abundance [9]. The technology drawbacks include a higher error rate at the base calling stage, lower throughput, and sensitivity to RNA degradation [4,10].

Numerous papers have been published comparing transcriptome profiles of normal and affected tissue to find disease biomarkers. At the same time, it turned out that the reproducibility of such studies at the level of individual genes is relatively poor [11,12]. In fact, even random gene expression signatures perform better than some published cancer outcome predictors [13]. Thus, instead of single gene analysis, researchers often characterize expression on the level of gene sets (pathways). Various methods were developed to assess pathway activation from transcriptomics data reviewed elsewhere [14,15]. The overall methodology of these tools is to estimate pathway perturbation scores, thus reducing dimensionality and noisy gene expression effect [16]. However, most methods for pathway activation borrow gene expression data from all samples in the cohort, which may lead to unstable scores in small data sets often encountered in practice. Instead, analysis of a single sample may detect individual-specific changes in transcriptome, thus paving the road to the truly personalized medicine. Recent review of methods that score molecular signatures and operate with single-subject transcriptome data can be found in Vitali et al. 2019 [17].

Several algorithms have been developed to infer activation scores for a single patient. One of them is an extension of the widely used Gene Set Enrichment Analysis method [18], so-called single-sample GSEA (ssGSEA) presented in [19]. In ssGSEA the score for a gene set is calculated as a sum of the differences between two weighted cumulative distribution functions of gene expressions inside and outside the set [20]. ssGSEA was used to develop an immune-based prognostic score for ovarian cancer [21], analyze the potential mechanisms causing differences in the immune response papillary thyroid cancer [22], and quantify the infiltration levels of immune cells in the bladder tumor microenvironment [23]. 

Another single sample algorithm for scoring perturbed pathways is singscore [24]. It has been successfully used for various applications, including investigation of transcriptional profiles associated with specific mutations in acute myeloid leukemia [25], detection of NK cell infiltration in metastatic cutaneous melanoma [26], and prediction of docetaxel response in breast cancer patients [27]. This rank-based method supports unidirectional (e.g., all genes are up-regulated) and bidirectional (with separate up and down-regulated genes) gene signatures and pathway scores are directly interpretable as a normalized mean percentile rank. Additionally, the singscore algorithm does not include the resampling step, eliminating the necessity to run it multiple times to ensure reproducibility.

In the present work, we attempted to analyze the performance of direct RNA-seq for single sample pathway scoring using MinION sequencer from Oxford Nanopore Technologies (ONT). We performed multiple RNA-seq replicates of HepG2 cell line using both long-read ONT and short-read Illumina technology. By focusing on protein-coding genes and transcripts, we assessed the reproducibility of ONT technical repeats and compared it with the Illumina platform in terms of the required number of replicates. Our results may be of interest for researchers planning to analyze gene abundances quantified via direct RNA-seq ONT sequencing.

## 2. Materials and Methods

### 2.1. Cells Preparation

The HepG2 cell line was obtained from Sigma-Aldrich (Merck KGaA). After thawing, the cells were grown in culture medium DMEM/F12 supplemented with 10% fetal bovine serum and 100 units/mL penicillin/streptomycin (all from Gibco, Amarillo, TX, USA) in a humidified CO_2_-incubator under standard conditions (5% CO_2_, 37 °C). The medium was exchanged every 3 days.

### 2.2. Sample and Library Preparation

Total RNA was isolated from HepG2 cells (5th passage, Sigma-Aldrich, St. Louis, USA) with an RNeasy Mini Kit (Qiagen, Hilden, Germany), quantified on a NanoDrop-1000 spectrophotometer (Thermo Fisher Scientific, Waltham, MA, USA) and its quality was assessed using a Bioanalyzer 2100 System (Agilent Technologies, Palo Alto, CA, USA). The RNA integrity numbers were 7.9 or higher for all RNA preparations. The mRNA extraction was conducted with a Dynabeads™ mRNA Purification Kit (Thermo Fisher Scientific, Waltham, MA, USA). The mRNA was quantified using a Qubit 4 fluorometer and a Qubit RNA HS Assay Kit (Thermo Fisher Scientific, Waltham, MA, USA). The mRNA preparations were either immediately used to prepare a sequencing library or frozen and stored at −80 °C until further use.

### 2.3. Transcriptome Profiling

Transcriptome data was obtained by high throughput paired-end sequencing using Illumina NovaSeq 6000 with read length equal to 100 bp. The TruSeq Stranded mRNA Library Prep Kit was used to prepare RNA libraries. All steps were carried out in accordance with the manufacturer’s protocol. Transcriptome profiling was performed in three replicates with a separate process of RNA extraction for each replicate. Samples of 30 μL total RNA with an average concentration of 300 ng/μL, were used for sequencing.

Nanopore sequencing was carried out on a MinION sequencer (Oxford Nanopore Technology, Oxford, UK) in 48-h single runs, using FLO-MIN106 flow cells and a Direct RNA sequencing kit (SQK-RNA002, ONT, Oxford, UK). The sequencing libraries were prepared following the manufacturer’s protocol and were either immediately sequenced or stored at −80 °C until use. The outputs varied between 1.4 and 2.9 Gb (0.96 to 2.6 million reads). The mean quality score for five technical replicates was equal to 10.2.

Raw sequencing data for both platforms were deposited to NCBI SRA (https://www.ncbi.nlm.nih.gov, accessed on 7 October 2021).

### 2.4. Data Analysis

For Illumina replicates, quality control was performed using FastQC [28]. Quality control for ONT data was carried out using the epi2me “Basic QC” pipeline (https://labs.epi2me.io/, accessed on 5 September 2021). Both ONT and Illumina replicates were aligned to the reference transcriptome Ensembl cDNA v.103. The resulting ONT fastq files were aligned using the long-read aligner Minimap2 using the “ax splice” mode [29]. Gene expression and isoform analysis for both platforms was performed using Salmon [30].

Expression of each transcript was quantified in transcript per million (TPM) units, giving relative abundance. Gene expression was calculated by summing all the TPMs of the corresponding transcripts.

Human genome annotation in the form of GTF file was downloaded from Ensembl resource (GRCh38, release 103). We limited our analysis to protein-coding transcripts by setting the following filters: type = ‘transcript’, gene_biotype = ‘protein_coding’, transcript_biotype = ‘protein_coding’ and tag = ‘basic’. We also did not include transcripts assigned to the non-standard chromosomes. This resulted in 60,740 protein-coding transcripts and 19,670 genes.

Two pathway scoring methods were employed, ssGSEA [19] and singscore [24]. We used ssGSEA implementation from the GSVA package [31], version 1.36.3, while for singscore corresponding R-package was utilized, version 1.8.0. Prior to analyses, low expressed genes with TPM less than 0.1 were removed. As a source of gene sets we used WikiPathways [32], a community-curated pathway database. Pathways containing less than 5 genes were removed, resulting in 526 pathways. Computational analyses and plots were performed using the R software environment (version 4.0) [33]. The R code can be found at [https://sourceforge.net/projects/ont-sysbiol/, accessed on 26 October 2021].

## 3. Results

**Figure 1:**
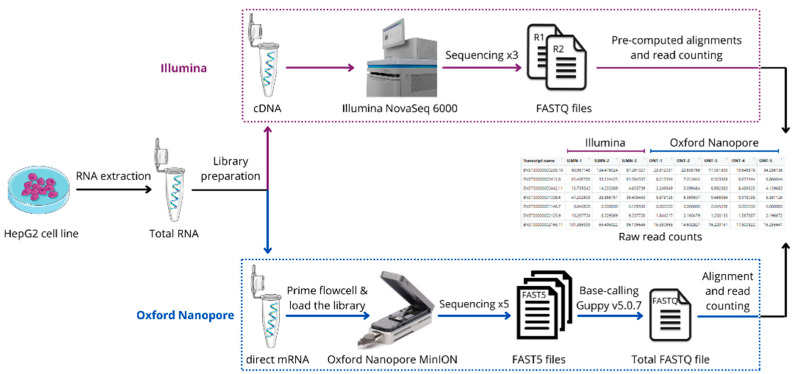
The overall workflow of the experiment. RNA was extracted from the HepG2 cell line and technical replicates were performed using Illumina NovaSeq 6000 (3 replicates) and Oxford Nanopore MinION (5 replicates). Expression of protein-coding transcripts was quantified using Salmon.

The overall workflow of the experiment and summary of data processing is presented in Figure 1. In brief, we performed multiple RNA-seq analyses for a single sample that originated from the HepG2 cell line. Five technical replicates were done using ONT MinION, and three technical replicates were done using Illumina NovaSeq 6000.

We performed basecalling with Guppy v5.0.7 [34] for ONT data. Only “pass” reads as designated by the tool were used for the subsequent analyses. Quality control for ONT data was carried out using the epi2me “Basic QC” pipeline (https://labs.epi2me.io/, accessed on 5 September 2021), while for Illumina data, we used FastQC [28]. Both ONT and Illumina reads were aligned to the reference transcriptome Ensembl cDNA v.103. Gene expression and isoform analysis was performed using Salmon [30].

The information about mapped reads, detected transcripts, and genes is provided in Table 1 for each sample. Three out of five ONT samples were comparable in terms of the total number of reads (approx. 1.8 million reads) except for ONT-1, which gave 2.4 million reads. Another outlier was ONT-3, which yielded only 0.95 million reads. We nevertheless decided to include this sample in the analysis as it may reflect various experimental difficulties occurring during the ONT RNA-seq experiment. For ONT data average read length was found to be 1255 ± 96 bp, while Illumina experiments were performed with each read length equal to 100 bp.

The obtained results show that our data is mostly concordant with other experiments using Illumina and ONT platforms. For example, a typical Illumina RNA-seq experiment yields ~30 million reads, while approximately one million reads per flow cell are obtained on average using MinION/GridION for direct RNA sequencing [35,36,37].

### 3.1. Intra- and Inter-Platform Reproducibility

**Figure 2:**
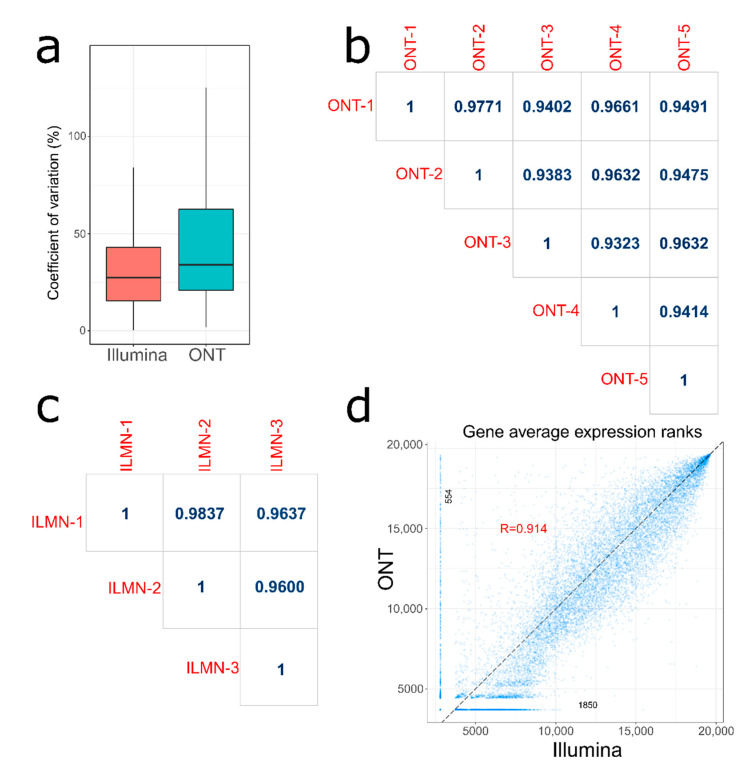
Comparison of intra- and inter-platform variation of gene quantification for Illumina and ONT. Data were restricted to genes that were observed in all replicates for each platform. Gene expression was measured in TPM units. (**a**) Distribution of coefficient of variation for both platforms. (**b**) Between replicates pairwise gene correlation matrix with Spearman coefficients for ONT platform. (**c**) Between replicates pairwise gene correlation matrix with Spearman coefficients for Illumina platform. (**d**) Correlation between average gene expression ranks for Illumina and ONT platform. Genes are represented by dots. Horizontal and vertical spurs indicate genes not detected by ONT (*n* = 1850) and Illumina (*n* = 554) platforms, respectively.

Next, we turned to the evaluation of the reproducibility of gene quantification for individual replicates. Data were restricted to genes that were observed in all replicates for each platform. The median coefficient of variation (CV) for the Illumina platform was found equal to 27.34%, while ONT showed a higher median CV equal to 33.93% (Figure 2a). The inter-platform correlation measured as Spearman coefficient between replicates was high both for ONT (average R = 0.952, Figure 2b) and Illumina (average R = 0.969, Figure 2c).

We also compared the agreement between two platforms in terms of gene ranking. For each gene, we calculated its average rank across three Illumina replicates and five ONT replicates. Pearson correlation between mean gene ranks was equal to R = 0.914 (Figure 2d). As expected, more pronounced cross-platform deviations can be seen for low expressed genes. Additionally, there were 554 genes not detected in any Illumina replicate and 1850 genes not detected in any ONT replicate. These genes are shown in Figure 2d as vertical and horizontal spurs.

Overall our results are consistent with other findings showing good inter- and intra-platform concordance [6,38]. However, as expected, the Illumina platform performed better in terms of reproducibility as compared to ONT.

### 3.2. ONT Replicates for Gene/Transcripts Identification

One of the drawbacks of the MinION sequencing device is the much lower throughput in terms of the read number as compared with the Illumina platform. This means that analysis of a single replicate detects only a fraction of all expressed transcripts [9,36]. Here we tried to estimate the number of ONT replicates required to detect the majority of protein-coding transcripts expressed in HepG2 cells. This can be of interest to researchers who would like to optimize the ONT-sequencing part of their experiments.

**Figure 3:**
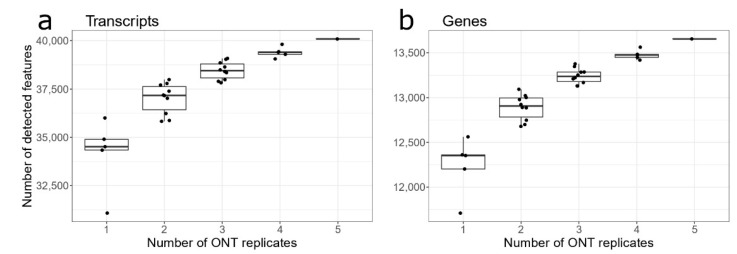
Total number of detected features for various number of replicates. Different combinations of experimental replicates ONT-1, …, ONT-5 were generated. Each transcript (**a**) or gene (**b**) was claimed to be expressed if it was detected in at least one replicate included in the combination.

We used the following approach to simulate the various number of replicates of the nanopore RNA-seq experiments. For each number of replicates, *n_rep_*, we took all possible combinations of experimental replicates. For example, for *n_rep_* = 4 we had the following combinations of ONT replicates: (1, 2, 3, 4), (1, 2, 3, 5), (1, 2, 4, 5), (1, 3, 4, 5) and (2, 3, 4, 5). Each transcript or gene was claimed to be expressed if detected in at least one replicate included in the combination. Results of the simulation are presented in Figure 3.

When changing the number of replicates, the sequencing performance was assessed by evaluating the number of expressed transcripts and genes. For a single ONT replicate on average, we detected 34,517 ± 557 protein-coding transcripts (median ± IQR) and 12,353 ± 158 protein-coding genes. For two ONT replicates, there were 37,172 ± 1196 transcripts and 12,906 ± 212 genes. As expected, the increased number of replicates quantified more expressed genes and transcripts, and for five replicates, we detected a total of 40,093 transcripts and 13,655 genes. The detection trend revealed a significant improvement in the number of quantified genes from one replicate to two replicates while adding more replicates gave relatively minor advantages. We may conclude that two ONT replicates seem to be a reasonable trade-off between the number of replicates and the number of quantified transcripts/genes.

### 3.3. ONT Replicates for Quantifying Pathway Activation

We evaluated whether gene abundances obtained via direct RNA sequencing with MinION technology are robust enough to be utilized for the prediction of activated pathways and genesets. To check it, we analyzed various datasets generated as combinations of ONT replicates. Data obtained from Illumina was used as a reference.

We utilized two popular methods to score molecular signatures (gene sets, pathways) in individual samples—singscore [24] and ssGSEA [31]. Both algorithms take gene expression in a sample and a collection of gene sets as input and return scores that measure the relative activation level of each gene set. Unlike many other methods, singscore and ssGSEA can be applied to single-subject transcriptomes.

WikiPathways was used as a collection of genesets [32]. Pathways containing less than five genes were removed, resulting in a total of 526 pathways. Both ONT and Illumina RNA-seq data were processed by filtering out low abundance genes with TPM less than 0.1.

**Figure 4:**
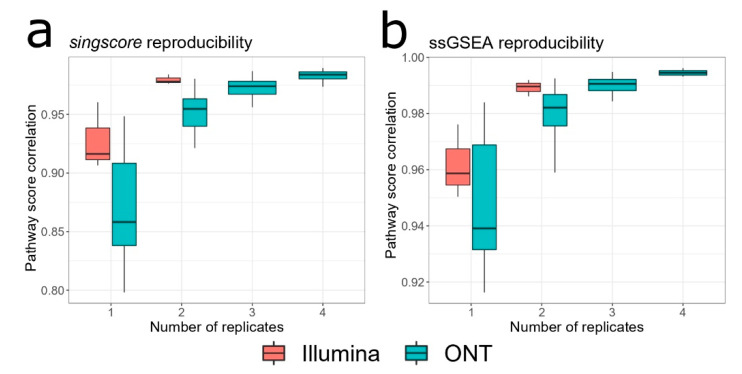
Pairwise correlation between pathway scores. Different combinations of experimental replicates ONT-1, …, ONT-5 and ILMN-1, …, ILMN-3 were generated. For every two combinations, pathway activation scores were estimated. Distribution of Spearman correlation coefficients between pathway scores using either singscore (**a**) or ssGSEA (**b**) algorithms was plotted for each number of replicates.

The technique for estimating the reproducibility of pathway activation was as follows. Similar to the methodology for gene/transcript quantification described earlier, for each number of replicates, *n_rep_*, we took all possible combinations of experimental replicates. For example for *n_rep_* = 4 we had the following combinations of ONT replicates: (1, 2, 3, 4), (1, 2, 3, 5), (1, 2, 4, 5), (1, 3, 4, 5) and (2, 3, 4, 5). For each combination, gene expression was summed, simulating increased sensitivity to quantify gene abundance. Then, for each pair of ONT replicates, e.g., (1, 2, 3, 4) and (1, 2, 3, 5), pathway scores were estimated via either singscore or ssGSEA. Spearman correlation between pathway scores for each pair was calculated, giving an estimate of pathway activation reproducibility. The whole process was repeated many times for all pairwise combinations of several ONT replicates. The same procedure was done for Illumina data. The simulation results are presented in Figure 4, which may be viewed as a key result of our study.

Obtained results for both pathway scoring algorithms generally coincide. As expected, the reproducibility of pathway activation scores improved with the increase in the number of replicates. For various pairs of single ONT replicates, the average correlation between singscore pathway scores was 0.858 ± 0.070 (median ± IQR), while for two replicates, it was equal to 0.955 ± 0.023. Further increase in the number of ONT replicates led only to relatively minor improvements in reproducibility: three replicates gave on average 0.974 ± 0.011 correlation between pathway scores. Results of the ssGSEA algorithm showed the same pattern. For different pairs of single ONT replicates, the average correlation between ssGSEA pathway scores was 0.939 ± 0.037, while for two replicates, it significantly increased and was equal to 0.982 ± 0.011. Average correlation coefficient when combining various three ONT replicates was equal to 0.991 ± 0.004.

Of note, reproducibility for Illumina data was found to be substantially better. For various pairs of single Illumina replicates, the average correlation between singscore pathway scores was 0.916 ± 0.027, while for ssGSEA the average correlation was equal to 0.959 ± 0.013. For various pairwise combinations of two Illumina replicates, the average correlation was 0.978 ± 0.004 for singscore and 0.990 ± 0.003 for ssGSEA. Overall using two Illumina replicates we achieved the same results in reproducibility of pathway activation scores as for three ONT replicates.

### 3.4. Confirmation of Results Using Published Data

The results obtained in the previous section were based on transcriptome profiling of the HepG2 cell line. However, these findings may not reproduce when studying other cell types. To check this, we searched NCBI SRA resource for similar datasets containing multiple replicates of either Illumina or MinION direct RNA-seq profiling of the same cell line except for the HepG2. For the HCT116 cell line, we found three replicates performed with Illumina NovaSeq 6000 (run ids SRR12698952, SRR12698953, SRR12698954) [39] and four direct RNA-seq replicates performed with MinION (run ids ERR6053047, ERR6053055, ERR6053056, ERR6053057) [40]. All ONT runs had more than 800,000 reads.

The same computational pipeline comparing pathway activation scores for various combinations of replicates was run. Simulation results for the HCT116 cell line are provided in Supplementary (Figure S1). For various pairs of single ONT replicates, the average correlation between singscore pathway scores was 0.910 ± 0.037. For two replicates it significantly increased to 0.962 ± 0.015, while adding the third replicate led to minor reproducibility improvements, 0.980 ± 0.006. Results of the ssGSEA algorithm followed the same pattern: for different pairs of single ONT replicates, the average correlation was 0.974 ± 0.008, while for two replicates, it increased up to 0.990 ± 0.003. For the HCT116 cell line Illumina data performance was also substantially better compared to the ONT platform: two Illumina replicates yielded the same reproducibility of pathway activation scores as three ONT replicates. Overall we conclude that our HepG2 transcriptome profiling results can be reproduced using published data on the HCT116 cell line.

## 4. Discussion

Nanopore RNA sequencing is becoming increasingly widespread due to its low cost, simplicity of library preparation, and potential to sense single-molecule modifications. Long reads make it possible to unambiguously identify expressed isoforms, thus increasing the accuracy of abundance estimates. The portability of the MinION device, coupled with its fast turnaround time, turns it into a promising tool to be used in clinical practice for early diagnosis, treatment, therapeutic monitoring, etc. [41].

Indeed, precision medicine aims at understanding disease mechanisms in single patients, and studying an individual’s transcriptome alterations may yield important theranostic results affecting the therapy strategy. Several bioinformatics tools have been developed to detect activated or repressed biological processes using gene expression data. However, most of them require either a large cohort of reference samples or use paired biopsies drawn from the same patient [17]. However, this scenario is not always available in real clinical applications. Hence, we focused on single sample methods (namely, ssGSEA [19] and singscore [24]) and assessed whether direct RNA-seq data generated by the MinION platform is suitable to be used for reproducible detection of activated pathways. Conventional transcriptome data generated by the Illumina platform for the same sample was used as a reference to compare with.

The key parameter for transcriptome profiling is the number of quantified transcripts. However, this value is of lesser importance for systems biology applications since algorithms operate only with genes ascribed to some pathways or gene sets. Thus, we ignored all non-coding transcripts and limited our analysis only to protein-coding genes. Using direct RNA-seq profiling of the HepG2 cell line, we detected on average more than 34,000 protein-coding transcripts assigned to more than 12,000 genes.

Our next step was to ensure that gene quantification for individual replicates is reproducible for intra- and inter-platform comparisons. We found that both Illumina and ONT showed good concordance between replicates, while the former platform performed better. This finding is in line with previous results [6,42]. The gene ranks correlation between both platforms was also relatively high, while there were genes whose expression was detected only by one of them.

An important parameter in planning transcriptome profiling experiments is the number of replicates. Since the MinION platform generates only a limited number of reads, this complicates the detection of less abundant transcripts for a single replicate [36]. We simulated the varying number of ONT replicates, estimating the total number of detected transcripts and genes. The obtained results indicated that two ONT replicates seem to be enough to detect expression for most of the transcripts and genes.

Using an exhaustive computational scheme for all replicate combinations and in agreement with the previous section, we found that two ONT replicates allow one to quantify the pathway activation with adequate reproducibility. This result was reproduced for both ssGSEA and singscore algorithms. As expected, a further increase in the number of ONT replicates led to improvements in pathway scoring reproducibility, while using two replicates seemed to be a reasonable trade-off. Another expected finding was the better performance of the Illumina NovaSeq platform as compared to the MinION device.

It should be noted that the field of nanopore-based transcriptomics is in active development. Direct RNA sequencing which does not utilize amplification steps has been only recently developed by ONT [6] and more work is needed to estimate this type of data in terms of reproducibility and biases [7,43]. To the best of our knowledge, our study is the first to apply system biology methods for single sample detection of activated pathways to the ONT direct RNA-seq data.

There are certain limitations of our analysis that we are aware of. Although we were able to reproduce our HepG2-based findings using published data on the HCT116 cell line, still this does not guarantee that it will hold for other cell types. There may be other methods for single-sample analysis which can potentially be utilized including FAIME [44]. However, the latter is microarray-oriented and operates on the normalized gene expression and not on more stable gene ranks. An arsenal of system biology methods has been developed to analyze differentially expressed genes, while there was a single condition in our experiment. So, it would be of interest to see the behavior of these algorithms for direct RNA-seq data. However, this comparison would likely require many ONT replicates since it has been estimated that from six to twelve Illumina RNA-seq replicates are required to detect significantly differentially expressed genes for all fold changes [45]. We believe that the aforementioned issues and limitations should be addressed in further research.

## 5. Conclusions

The aim of our study was to evaluate the performance of direct RNA-seq via ONT MinION platform for systems biology applications, namely for single-sample quantification of pathway activation scores. In brief, we found that at least two experimental replicates are required to obtain reproducible results. We hope that these findings and developed methodology may be of interest to researchers planning their ONT direct RNA-seq experiments.

## Figures and Tables

**Table 1:** Information about mapped reads and numbers of detected protein-coding transcripts and genes for each sample.

Sample	Num Total Reads	Average Read Length	Num Mapped Reads	Num Expressed Protein-Coding Transcripts	Num Expressed Protein-Coding Genes
ONT-1	2,416,117	1174	2,329,641	36,001	12,562
ONT-2	1,813,263	1212	1,175,355	34,898	12,361
ONT-3	956,446	1381	914,186	31,078	11,710
ONT-4	1,807,655	1176	1,630,381	34,341	12,203
ONT-5	1,875,286	1333	1,799,768	34,517	12,353
ILMN-1	59,789,684	100	53,499,563	30,552	13,961
ILMN-2	95,048,978	100	86,520,672	31,954	14,104
ILMN-3	54,075,176	100	46,702,470	28,993	13,972

## Data Availability

All raw and processed data files were uploaded to the NCBI SRA (https://www.ncbi.nlm.nih.gov, accessed on 7 October 2021), accession PRJNA765908. Datasets ONT-1, ONT-2, ONT-3, ONT-4, ONT-5 and ILMN-1, ILMN-2, ILMN-3 can individually be downloaded via accession numbers SRR16071311, SRR16071318, SRR16071317, SRR16071316, SRR16071315, SRR16071314, SRR16071313, SRR16071312, respectively.

## Supplementary Materials

The following are available online at https://www.mdpi.com/article/10.3390/biology10111131/s1, Figure S1: Pairwise Spearman correlation between pathway scores for different combinations of experimental replicates using published data for HCT 116 cell line.
