# Supplementary material for: Oxford Nanopore MinION Direct RNA-Seq for Systems Biology
Source: Biology (Basel). 2021 Nov 4;10(11):1131. doi: 10.3390/biology10111131 (PMC8615092; doi:10.3390/biology10111131)
Supplement: Supplementary file 1 [file biology-10-01131-s001.zip › biology-1434443-supplementary.pdf]

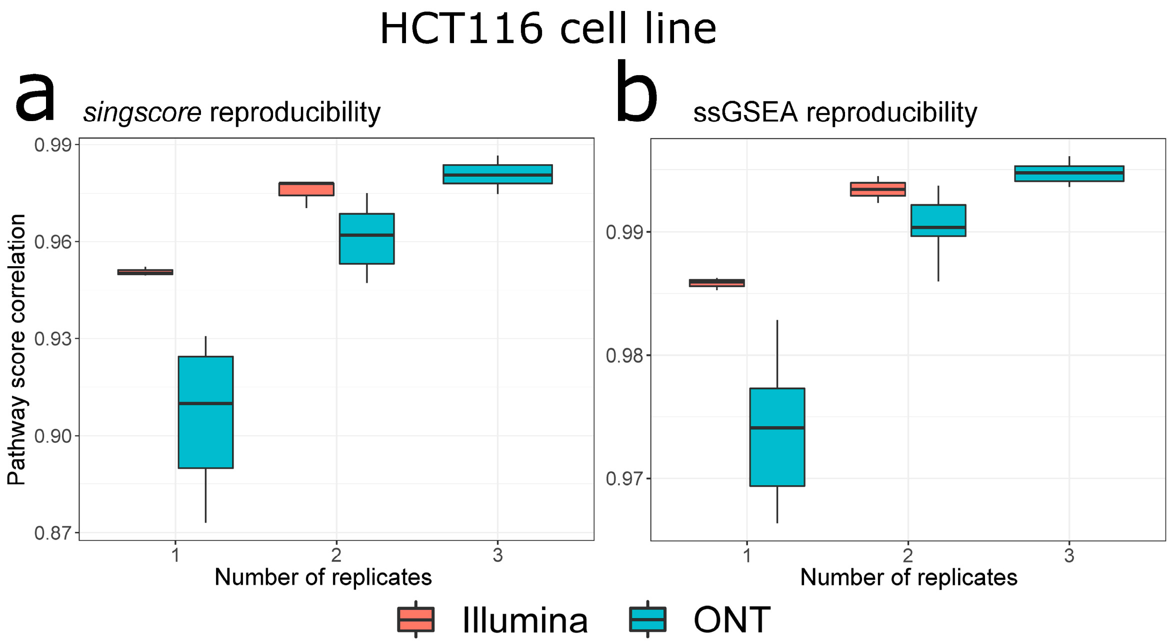

Figure S1. Pairwise Spearman correlation between pathway scores for different combinations of experimental replicates using published data for HCT 116 cell line.
